# Assessment of Bacterial Community Composition and Dynamics in Alfalfa Silages With and Without *Lactobacillus plantarum* Inoculation Using Absolute Quantification 16S rRNA Sequencing

**DOI:** 10.3389/fmicb.2020.629894

**Published:** 2021-01-26

**Authors:** Fengyuan Yang, Shanshan Zhao, Yuan Wang, Xiaomiao Fan, Yanping Wang, Changsong Feng

**Affiliations:** ^1^Henan Provincial Key Laboratory of Ion Beam Bio-engineering, School of Agricultural Science, Zhengzhou University, Zhengzhou, China; ^2^Henan Provincial Key Laboratory of Ion Beam Bio-engineering, College of Physics, Zhengzhou University, Zhengzhou, China; ^3^Institute of Animal Husbandry and Veterinary Science, Henan Academy of Agricultural Sciences, Zhengzhou, China

**Keywords:** absolute quantification 16S-seq, relative quantification 16S-seq, alfalfa silage, *Lactobacillus plantarum*, bacterial community

## Abstract

Relative quantification 16S-seq (RQS) has drawn deeper insights into bacterial community compositions in silage. However, it provides no information on dynamics of the total amount of bacterial DNA through the ensiling process and across different treatments. In this study, bacterial compositions in alfalfa silage with and without *Lactobacillus plantarum* inoculation after 10 and 60days of ensiling were investigated using absolute quantification 16S-seq (AQS), and bacterial composition and its interaction with fermentation properties of silage indicated by AQS and RQS were compared. Variation in total bacterial DNA amounts across different treatments and ensiling periods was illustrated by AQS. AQS indicated higher bacterial richness indices and closer correlations of these indices with fermentation properties than RQS *via* spearman’s correlation analyses, as well as more taxa with significance on bacterial abundance *via* lefse analyses. In conclusion, AQS effectively illustrated the dynamics of bacterial communities during the ensiling process.

## Introduction

Ensiling has become a global practice for forage preservation (Eikmeyer et al., 2013). It is an anaerobic microbial-based fermentation process, during which lactic acid bacteria (LAB) dominate the bacterial community and produce lactic acid (LA) for pH decline and undesirable microorganism inhibition. Alfalfa (*Medicago sativa L.*) is a widely cultivated and economically valuable pasture plant rich in protein [229g crude protein (CP)/kg on a dry matter (DM) basis, Ogunade et al., 2016; 277g CP/kg DM, Yang et al., 2019] and is an important forage crop used for ensiling worldwide (Dunière et al., 2013). However, alfalfa is in fact hard to ensile owing to its high buffering capacity and lack of sufficient water-soluble carbohydrates (WSCs). *Lactobacillus plantarum* (*L. plantarum*) is the most commonly used bacterial inoculant in forage ensiling studies (Oliveira et al., 2017) due to its enhancement in silage acidification and good adaption to low pH environment.

The microbial community plays an important role in the ensiling process. The development of PCR-based techniques enables us to define the microbial communities more accurately, and recent studies applying high through put sequencing technology have illustrated the dynamics and compositions of relative abundance of microorganism groups in alfalfa silage during ensiling process (Guo et al., 2018; Ogunade et al., 2018; Yang et al., 2019, 2020). However, the relative quantitative analyses cannot reflect the true absolute abundance of microorganism groups in a sample. For example, the increase in the relative abundance of a certain group of microorganisms may not reflect the increase of its absolute abundance. Instead, it may be linked to the decrease in the absolute abundance of other microorganisms. Thus, disregarding of the absolute abundance of microbial community in conventional 16S amplicon sequencing technology based on relative quantitative analyses may lead to incomprehensive interpretations (Props et al., 2017; Vandeputte et al., 2017). Although quantitative real-time polymerase chain reaction (qPCR) can perform absolute quantitative analyses of microorganism groups, it requires a high specificity of primers, resulting in conventional qPCR hardly applicable to the absolute quantification of microorganisms in complex environmental samples (Tkacz et al., 2018). In addition, humic acids in the silage samples will inhibit polymerase chain reaction by inhibiting the activity of enzymes, thereby affecting the accuracy of qPCR quantitative results of bacterial copy number.

Recent studies based on relative quantitative analyses document a larger variation in the microbiota in alfalfa silages than in cereal silages (McAllister et al., 2018). Additionally, a number of genera have been identified in alfalfa silage samples, while their roles and dynamics during ensiling were still at little understood (Ogunade et al., 2018). Recently, Tkacz et al. (2018) developed an absolute quantitation of different microbial taxa in highly complex samples using synthetic chimeric DNA spikes, and successful practice has been performed on comparison of differentially abundant taxa across soil samples (Jiang et al., 2019). This absolute quantification 16S-seq (AQS) method has been confirmed to uncover the comprehensive dynamics of bacterial communities (Jiang et al., 2019).

The objective of this study is to get deeper insights into the dynamics of bacterial community during alfalfa ensiling process and to evaluate the necessity of evaluating the total amount of bacterial DNA in silage bacterial community analyses. The changes in bacterial community compositions at different ensiling stages including the impact of *L. plantarum* inoculation were determined by AQS.

## Materials and Methods

### Silage Preparation

Alfalfa was harvested at early bloom stage on May 9, 2019 from Zhengzhou, Henan Province (temperate monsoon climate, 34.76°N, 113.65°E, altitude 110.4m above sea level), and wilted to a DM of 371.57±2.39g/kg fresh weight (FW). The wilted alfalfa was chopped to a theoretical length of 2cm using a crop cutter. *Lactobacillus plantarum* A345 was used as an additive for alfalfa silage. It was an alfalfa epiphytic isolated from Shanxi, China. Cultivated strain was centrifuged, and the precipitate was mixed with sterilized water to an OD_600_ of 0.8.

Approximately 500g for each of replicates of the chopped alfalfa were treated with the following: sterilized water control (CK); and 1 × 10^6^cfu/g of *L. plantarum* A345 (LP). A total of 12 bags (2 treatments × 2 ensiling periods × 3 replicates) were vacuumed at ambient temperature (21–27°C). Three bags for each treatment were opened for analyzing the pH, fermentation products, and bacterial community after 10 and 60days of ensiling, respectively.

### Analyses of pH and Fermentation Products

For pH and organic acid determination, fresh samples of silage (10g) were diluted with 90ml of sterilized water, shaked at 180r/min for 2h, and filtered through a 0.45-μm membrane. The pH was immediately measured with a glass electrode pH meter (Mettler Toledo CO., Ltd., Greifensee, Switzerland). The organic acid contents were determined using high-performance liquid chromatography (Waters, Inc., Milford, MA, United States) following the procedure described by Zhao et al. (2020). The ammoniacal nitrogen (NH_3_-N) concentration was determined using Berthelot colorimetry (Broderick and Kang, 1980).

For DM determination, fresh samples were dried in an oven at 65°C for 48h (AOAC, 1990). The dried materials were pulverized to pass through a 1-mm screen with a laboratory knife mill (FW100, Taisite Instrument Co. Ltd., Tianjin, China). The WSCs were determined using anthrone colorimetry (Murphy, 1958).

### Analyses of Bacterial Community

Each of the 12 samples (2 treatments × 2 ensiling periods × 3 replicates; 10g) was mixed with 100ml of sterile phosphate buffer saline (pH 7.2) by vigorous shaking at 180r/min for 2h. The mixture was filtered through four layers cheesecloth and the liquor was then centrifuged at 10,000r/min for 10min at 4°C. The precipitate was resuspended in 1ml of sterile phosphate buffer saline. The liquor was centrifuged at 12,000r/min for 10min at 4°C to collect microbial pellet. Total DNAs were extracted using a Bacterial DNA Kit D3350-02 (Omega Biotek, Norcross, GA, United States). The PCR amplifications of the V3–V4 regions of the bacterial 16SrDNA gene were performed using Primer F (Illumina adapter sequence 1+ CCTACGGGNGGCWGCAG) and Primer R (Illumina adapter sequence 2+ GACTACHVGGGTATCTAATCC). The PCR products were extracted from a 2% agarose gel and purified using AgencourtAMpure XP nucleic acid purification magnetic beads to obtain an original library of samples. The general procedure of AQS was outlined by Smets et al. (2016) and Tkacz et al. (2018). Briefly, synthetic chimeric DNA spikes were designed with variable regions lacking identity to nucleotide sequences deposited in public databases. This allows robust tracing of spike-in reads in 16S-seq data from any microbial samples. With known amounts of synthetic chimeric DNA spikes added to the samples, these spikes could be used as internal standards for absolute quantification. Nine different synthetic chimeric DNA spikes with four different concentrations (10^3^, 10^4^, 10^5^, and 10^6^ of copies of internal standards) were added to the sample DNA pools. The amplicon sequencing of 16SrDNA was conducted using the Miseq platform (Genesky Bio-Tech Co. Ltd., Shanghai, China) after the purification and quantification of the PCR products.

All the raw reads were checked using FLASH2 (version 2.2.00), and low quality sequences (quality scores below 20) were discarded according to the QIIME quality control process (version 1.7.0). Operational taxonomic units were clustered using Uparse (version 7.0.1001) at 97% similarity. For AQS, the synthetic chimeric DNA spikes were filtered out and reads were counted. The copy numbers were then rectified based on ribosomal RNA operons (rrn) DataBase using the procedure described by Stoddard et al. (2015) and Wu et al. (2017). The analyses of taxonomy assignment of representative sequences was performed using the Ribosome Database Project (Cole et al., 2009). The sequence data had been submitted to the GSA database under accession number CRA003223.

### Statistical Analyses

The pH and fermentation products’ data were analyzed by general liner model using IBM SPSS version 21.0 (SPSS Inc., Chicago, IL, United States). For bacterial community analyses, samples with same treatment and same ensiling time were treated as a group (e.g., 10d_CK). Bacterial community compositions of the four groups (2 treatments × 2 ensiling periods) were compared. The alpha diversity indices of bacterial communities were calculated using mothur (version 1.9.1) and analyzed using Kruskal-Wallis test performed by R (version 3.4.3). Principal coordinate analyses (PCoA) was performed by R (version 3.4.3) based on Bray-Curtis measure. The Lefse analyses was performed using python (version 2.7.14). Correlation analyses of bacterial genera and fermentation properties was performed using Spearman’s rank correlation coefficient.

## Results

### Characteristics of Alfalfa Material Prior to Ensiling

Alfalfa was harvested at early bloom stage and wilted to a DM of 372g/kg FW. The wilted alfalfa had a pH value of 6.57, and a WSC concentration of 7.61%DM. The WSC concentration slightly decreased after wilting (8.21%DM prior to wilting). No formation of organic acids or NH_3_-N was detected.

### Effects of *Lactobacillus plantarum* on Fermentation Properties of Alfalfa Silage

Effects of treatment of *L. plantarum* (T), days of ensiling (D), and their interactions on alfalfa silage fermentation properties are listed in Table 1. The pH value and NH_3_-N concentration in alfalfa silage were significantly affected by inoculation, ensiling period, as well as their interactions (*p* < 0.05). *Lactobacillus plantarum* inoculation also affected LA concentration and lactic acid to acetic acid ratio (LA/AA) in alfalfa silage (*p* < 0.05). Ensiling period affected the pH value, WSC and NH_3_-N concentrations in silage (*p* < 0.05). And the fermentation properties were all affected by their interactions (*p* < 0.05).

**Table 1:** Effects of treatment (T), ensiling days (D), and their interactions (T × D) on fermentation properties in alfalfa silage.

Items^1^	Treatment	Ensiling days (d)		SEM	Significance^2^		
		10	60		T	D	T × D
pH	CK	6.13^bB^	5.31^bA^	0.06	*p* < 0.01	*p* < 0.01	*p* < 0.01
	LP	4.75^a^	4.60^a^				
Lactic acid (g/kg DM)	CK	9.11^aA^	32.44^B^	5.04	*p* < 0.01	NS	*p* < 0.01
	LP	55.42^bA^	41.47^B^				
Acetic acid (g/kg DM)	CK	13.46^aA^	15.32^bB^	0.3	NS	NS	*p* < 0.01
	LP	15.04^b^	14.29^a^				
Lactic acid/acetic acid	CK	0.63^aA^	2.12^B^	0.35	*p* < 0.01	NS	*p* < 0.05
	LP	3.68^b^	2.90				
WSC (g/kg DM)	CK	20.08^bB^	4.36^aA^	1.42	NS	*p* < 0.01	*p* < 0.01
	LP	12.63^a^	8.96^b^				
NH_3_-N (g/kg DM)	CK	2.50^bA^	4.66^bB^	0.17	*p* < 0.01	*p* < 0.01	*p* < 0.01
	LP	1.17^aA^	1.86^aB^				

Means of three observations. Values with different capital letters in a row indicate significance over ensiling days (*p* < 0.05). Values with different small letters in a column indicate significance over treatments (*p* < 0.05).

1 DM, dry matter; NH_3_-N, ammoniacal nitrogen; WSC, water soluble carbohydrate.

2 NS, not significant.

Inoculation with *L. plantarum* accelerated the LA fermentation process, indicated by the rapid pH decline, LA accumulation and WSC consumption, as well as the high LA/AA in 10-day silage (*p* < 0.05). At 60 days, the inoculated silage had a lower pH value, higher LA concentration, and better NH_3_-N inhibition comparing with the CK group (*p* < 0.05). Neither propionic acid nor butyric acid content was detected in alfalfa silage.

### Dynamics of Bacterial Communities in Silage Illustrated by AQS and RQS

**Figure 1:**
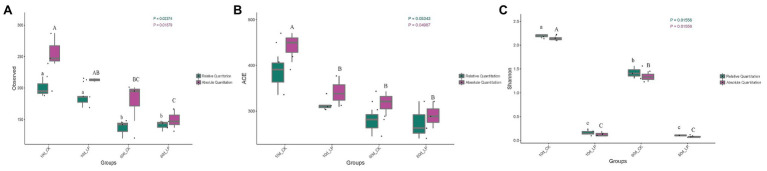
Box-plots of observed bacterial species **(A)**, ACE **(B)** and Shannon indices **(C)** of bacterial communities in alfalfa silage. Values with different capital letters indicate significance over groups illustrated by absolute quantification 16S-seq (AQS; *p* < 0.05). Values with different small letters indicate significance over groups illustrated by Relative quantification 16S-seq (RQS; *p* < 0.05). CK, control; LP, inoculated with *L. plantarum* A345. The numbers ahead of CK and LP stand for ensiled days of silage. Observed, observed bacterial species.

High-throughput analyses determined the bacterial community compositions in alfalfa silage, and the valid sequences were clustered into 1,029 operational taxonomic units based on a 97% sequence identity. Gene copies in per ng DNA of the samples were calculated by standard curve formula based on a 99% coefficient of determination (*R*^2^). The bacterial richness indices represented by observed species and ACE richness estimate index, and the bacterial diversity represented by Shannon index were shown in Figures 1A–C. Richness of bacterial community in silage decreased after ensiling for 60days compared with 10-day silage (Figures 1A,B). AQS illustrated higher bacterial richness indices than Relative quantification 16S-seq (RQS). And AQS indicated a significant variance on ACE index in 10d_CK silage over other groups (*p* < 0.05), while RQS failed to indicate the variance (*p* = 0.05343). AQS and RQS both clearly revealed the diversity of bacterial community among the samples (Figure 1C). Diversity of bacterial community decreased with *L. plantarum* inoculation (*p* < 0.05). Shannon index in CK group decreased at 60-day compared to 10-day silage (*p* < 0.05).

**Figure 2:**
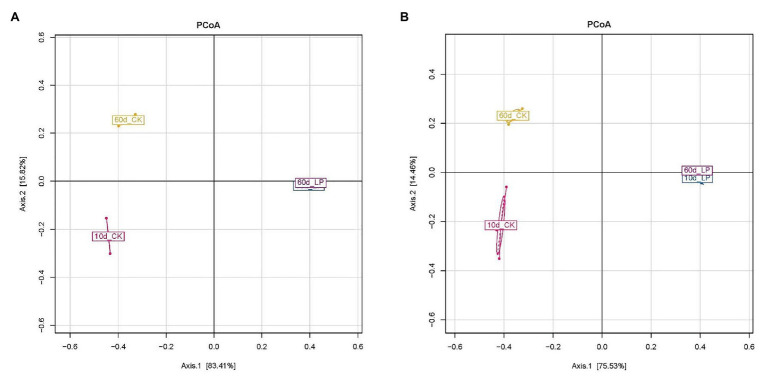
Principal Coordinate Analysis of bacterial communities in alfalfa silage based on the results illustrated by RQS **(A)** and AQS **(B)**. CK, control; LP, inoculated with *L. plantarum* A345. The numbers ahead of CK and LP stand for ensiled days of silage.

Variation within the bacterial community was reflected by PCoA (Figures 2A,B). Divisions in the plots representing silage with and without inoculation indicated that the distribution of the bacterial community was shifted by *L. plantarum* inoculations. The clear separation between bacterial communities of 10- and 60-day silage in the CK group indicated a shift between the two periods in naturally ensiled silages. The distribution of the bacterial communities among the three replications within 10d_CK exhibited by AQS was more separated compared to those exhibited by RQS. Meanwhile, AQS exhibited a slightly larger variation between the 10d_LP and 60d_LP group than RQS.

**Figure 3:**
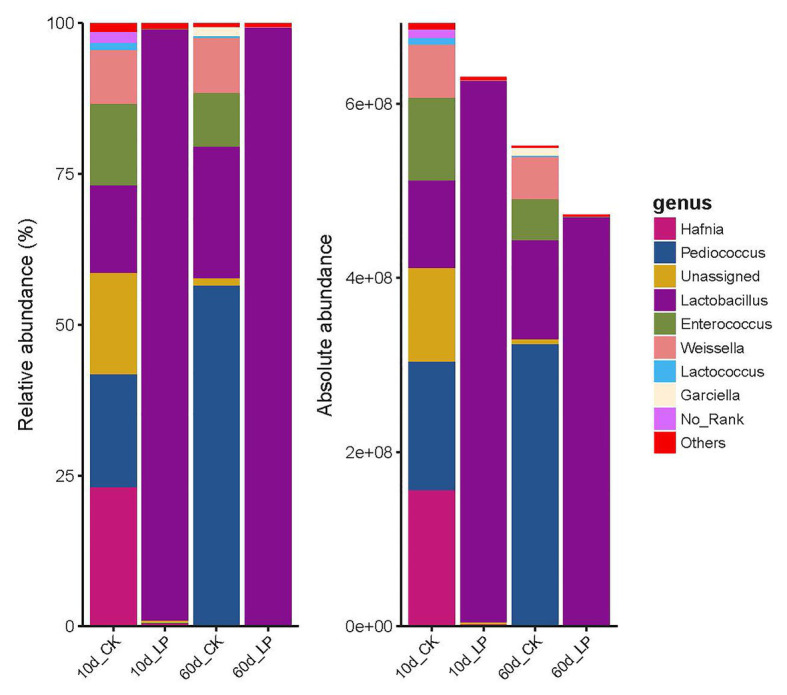
Barplots of bacterial communities in alfalfa silage illustrated by RQS and AQS. CK, control; LP, inoculated with *L. plantarum* A345. The numbers ahead of CK and LP stand for ensiled days of silage.

Bacterial community structure on genus level indicated by RQS and AQS are shown in Figure 3. *Lactobacillus plantarum* inoculation altered the abundance of major bacteria involved in LA fermentation of alfalfa silage. *Lactobacillus* was the predominant genus of the bacterial community in the inoculated group at both periods (97.97% at 10days and 99.09% at 60days). While *Pediococcus* was the predominant genus in 60-day silage without inoculation (56.53%), followed by *Lactobacillus* (21.74%), *Weissella* (9.14%), and *Enterococcus* (8.90%). At 10days, silage in the CK group exhibited a complex bacterial community composition, including *Hafnia* (23.09%), *Pediococcus* (18.76%), Unassigned (16.77%), *Lactobacillus* (14.51%), *Enterococcus* (13.51%), and *Weissella* (8.98%). This is consistent with the high Shannon index in 10d_CK group (Figure 1C). Dynamics and effects of *L. plantarum* inoculation on the total amount of bacterial DNA were indicated by AQS. Inoculation exhibited a reducing effect on the total amount of bacterial DNA compared with the CK group (6.30×10^8^ copies/ng DNA vs. 6.93×10^8^ copies/ng DNA at 10days and 4.73×10^8^ copies/ng DNA vs. 5.52×10^8^ copies/ng DNA at 60days). Absolute abundance of *Lactobacillus* reduced in the inoculated group at 60days compared with in 10days silage (4.69×10^8^ copies/ng DNA at 60days and 6.22×10^8^ copies/ng DNA at 10days).

**Figure 4:**
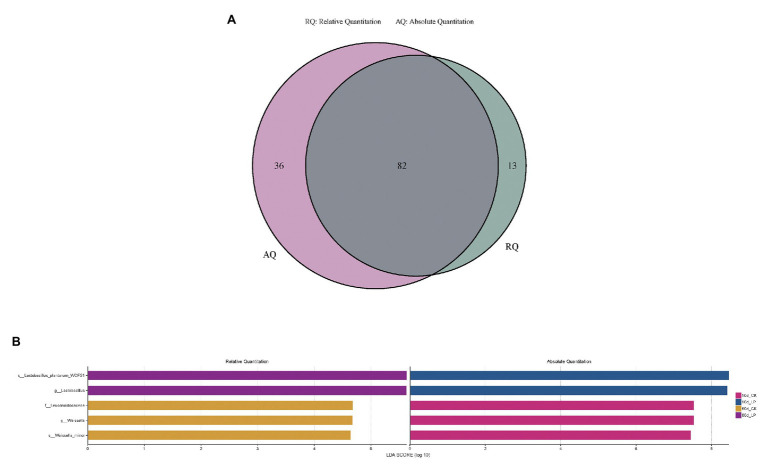
Comparison of microbial variations using LefSe analysis illustrated by RQS and AQS in Venn **(A)** and barplot **(B)** form. CK, control; LP, inoculated with *L. plantarum* A345. The numbers ahead of CK and LP stand for ensiled days of silage.

LefSe analysis identified the high-dimensional biomarkers in alfalfa ensiling microbiota among each treatment (Figures 4A,B). Bacterial groups at different taxon levels with significance on bacterial abundance among the treatments illustrated by AQS and RQS also varied. Eighty-two bacterial groups with significance between the treatments were both illustrated by AQS and RQS. While 36 bacterial groups with significance were only illustrated by AQS, 13 bacterial groups were only illustrated by RQS (Figure 4A). On genus level, AQS illustrated significance of *Lactobacillus* and *Weissella* on bacterial abundance among each treatment in 10d_LP and 10d_CK group, respectively. While RQS illustrated their significance in 60d_LP and 60d_CK group (Figure 4B).

### Correlation Analyses of the Bacterial Community With Fermentation Properties

Correlation of Alpha diversity indices with fermentation properties in alfalfa silage is shown in Table 2. The Shannon index was positively correlated with pH and NH_3_-N and negatively correlated with LA and LA/AA (*p* < 0.05). RQS and AQS illustrated similar correlations of the Shannon index with fermentation properties, as the Shannon indices indicated by AQS and RQS were similar (Figure 1C). AQS illustrated positive correlations of observed bacterial species and ACE index with pH in silage (*p* < 0.05). These correlations were not exhibited by RQS, because it did not indicate the reduction in the total amount of bacterial DNA at 60days. The WSC had negative correlations with observed bacterial species and ACE index (*p* < 0.05).

**Table 2:** Spearman’s correlation analyses of alpha diversity indices with fermentation properties illustrated by RQS and AQS.

		pH		LA		AA		LA/AA		NH_3_-N		WSC	
		*r*	*P*	*r*	*P*	*r*	*P*	*r*	*P*	*r*	*P*	*r*	*P*
Observed^1^	RQS	0.517	0.089	−0.333	0.291	−0.350	0.266	−0.298	0.347	−0.231	0.471	0.918	0.000
	AQS	0.713	0.012	−0.392	0.207	−0.098	0.766	−0.357	0.254	−0.056	0.869	0.816	0.001
ACE^2^	RQS	0.462	0.134	−0.406	0.190	−0.406	0.193	−0.364	0.244	−0.098	0.766	0.809	0.001
	AQS	0.671	0.020	−0.539	0.070	−0.126	0.700	−0.518	0.084	0.112	0.733	0.704	0.011
Shannon	RQS	0.965	0.000	−0.781	0.003	−0.084	0.800	−0.774	0.003	0.601	0.043	0.403	0.194
	AQS	0.965	0.000	−0.781	0.003	−0.084	0.800	−0.774	0.003	0.601	0.043	0.403	0.194

1 Observed, observed bacterial species.

2 ACE richness estimate.

**Figure 5:**
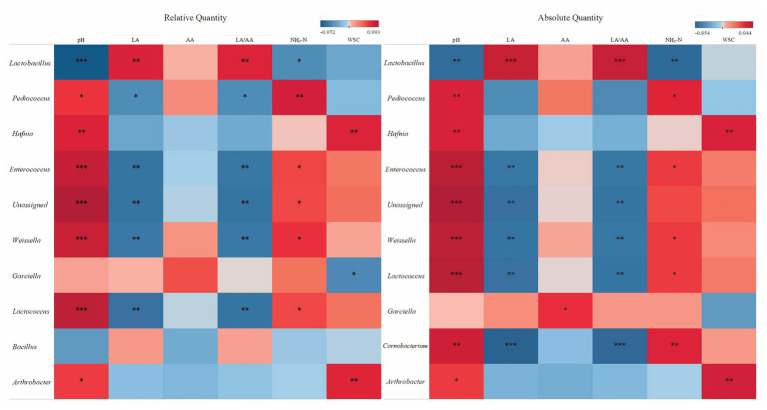
Spearman correlation heatmap of abundance of the top 10th abundant bacterial genera and fermentation properties in alfalfa silage illustrated by RQS and AQS. ^*^p < 0.05; ^**^p < 0.01; ^***^p < 0.001.

Spearman’s correlations further illustrated the relationships between bacterial genera and silage properties (Figure 5). *Lactobacillus* correlated positively with LA and LA/AA and correlated negatively with pH and NH_3_-N (*p* < 0.05). *Pediococcus*, *Enterococcus*, *Weissella*, and *Lactococcus* exhibited positive correlations with pH and NH_3_-N (*p* < 0.05), and these genera also exhibited negative correlations with LA and LA/AA excluding of *Pediococcus* (*p* < 0.05). *Hafnia* and *Arthrobacter* correlated positively with pH and WSC (*p* < 0.05). Correlation of *Garciella* with fermentation properties illustrated by RQS and AQS varied. RQS illustrated a negative correlation between *Garciella* and WSC (*r* = −0.62; *p* < 0.05), and a poor correlation of *Garciella* with AA (*p* > 0.05). While AQS illustrated a positive correlation between *Garciella* and AA (*r* = 0.66; *p* < 0.05). AQS illustrated positive correlations of *Carnobacterium* with pH and NH_3_-N and negative correlations with LA and LA/AA (*p* < 0.05).

## Discussion

In the recent years, application of RQS in forage ensiling studies has brought us a deeper insight into the microbial community and its interaction with fermentation properties and metabolites in silage samples (Xu et al., 2019; Wang et al., 2020). RQS effectively illustrated composition of bacterial community in a single sample. But solely application of RQS might be misleading when exploring bacterial community dynamics through the ensiling process or across multiple treatments, since fluctuations in absolute abundance of a certain microbial group may not cause a significant change on relative abundance of the taxon when the total abundance of bacterial community is not fixed (Jiang et al., 2019). The current study explored the dynamics of bacterial community at different ensiling periods, and effects of *L. plantarum* inoculation on absolute abundance of microorganisms in alfalfa silage with the application of AQS.

### Characteristics of Wilted Alfalfa

Characteristics of forage affect fermentation quality of silage (Guan et al., 2018). For low-DM crops like wet grasses and legumes, it is recommended to wilt them to a DM content of 300–400g/kg FW to prevent effluent production (Dunière et al., 2013). In this study, The DM of alfalfa material was wilted to an appropriate level (372g/kg FW). The WSC concentration decreased after wilting, which was consistent with the results reported by Tao et al. (2017) and Agarussi et al. (2019). The WSC content of wilted alfalfa was higher than 5%DM, which was sufficient for adequate fermentation during ensiling (Ni et al., 2018).

### Fermentation Quality of Silage Improved With *Lactobacillus plantarum* Inoculation

Inoculation of *L. plantarum* improved fermentation quality of alfalfa silage. The NH_3_-N is considered as a representation of proteolytic activity (Scherer et al., 2015). The inhibiting effect on NH_3_-N accumulation of *L. plantarum* inoculation suggested an enhancement on protein preservation during ensiling. Plant proteolytic enzymes was thought to be a typical cause for NH_3_-N accumulation (Kung and Shaver, 2001). Tao et al. (2012) reported that most plant proteolytic enzymes in alfalfa silage showed greater activities at pH 5.0–6.0, which might be an explanation for the reduction in NH_3_-N formation in the inoculated group. Similar effects of *L. plantarum* inoculant in alfalfa silage were also reported in the former studies (Ogunade et al., 2016; Yang et al., 2019). Neither propionic acid nor butyric acid content was detected in alfalfa silage in the current study, which is desirable, because their presence was thought to be a waste of metabolic energy (Dong et al., 2020) and an indication of spoilage microorganisms’ presence and activity (Dunière et al., 2013). AA concentration in silage was also at desirable nutritional level (<30g/kg DM) (Kung and Shaver, 2001; Ni et al., 2017). LA concentration decreased at 60days compared to 10-day silage in the inoculated group. This was consistent with the results reported by Agarussi et al. (2019) that LA concentration in inoculated silage reduced at 56days compared to 7-day silage. It has been well documented that the optimum pH value for stable silage is to below 4.2 (Wang et al., 2017), while this optimum pH value was hardly reached in alfalfa silage with LAB inoculation (Guo et al., 2018; Agarussi et al., 2019). In this study, although the WSC concentration in fresh material was considered sufficient for adequate fermentation, pH of 60-day silage in the LP group (4.60) was still above optimum level. This might due to the relatively high buffering capacity of alfalfa (548.81mE/kg DM, Tao et al., 2017; 450mE/kg DM, Zhang et al., 2017) compared to the cereal forage. Addition of extra sucrose might aid in reaching the optimum pH level. Zheng et al. (2017) reported a pH value of 4.03 in the direct-cut alfalfa silage with 2% sucrose addition along with 1 × 10^6^cfu/g *L. plantarum* inoculant.

### AQS Illustrated Comprehensive Dynamics of Bacterial Community in Silage

AQS accurately illustrated the dynamics in absolute abundance of bacterial community in alfalfa silage using synthetic chimeric DNA spikes, and uncovered more sequencing information analyzing alpha and beta diversities of bacterial communities than RQS. Dong et al. (2020) reported a reduction in richness of bacterial community through the ensiling process in *Broussonetia papyrifera* and perennial ryegrass mixed silage. Similarly, bacterial richness indices in our study reduced at 60days compared with the 10-day alfalfa silage. Diversity of bacterial community decreased with *L. plantarum* inoculation. Mendez-Garcia et al. (2015) reported that a low pH was the main factor underlying the limited microbial diversity in acid environments. This might explain the decreased bacterial diversity in silage with *L. plantarum* inoculant, as the inoculation significantly reduced the pH level in alfalfa silage (*p* < 0.05). Similar effect of LAB inoculants on reducing bacterial diversity was also reported by Ni et al. (2017) and Ogunade et al. (2018) in alfalfa and soybean silage, respectively.

AQS illustrated deeper and more accurate information in bacterial community than RQS. This was due to rarefaction of the number of reads of samples in RQS analyses. During the RQS analyses, the number of reads of samples will be randomly drawn to equal level, in order to avoid deviations in analyses due to different data sizes of samples. Generally, the minimum number of reads in sequencing samples is selected as the base, that is, the number of reads of all samples will be equally drawn to this value. Thus, this process makes the RQS analyses disregard the variation in total bacterial DNA amount across samples and lose the information of some reads in the samples. On contrary, AQS calculates the copy number of each microorganism in the sample to achieve absolute quantification, so all the reads of the samples are preserved. This might explain the higher richness indices, larger variation among samples in PCoA and variation in results of Lefse analyses illustrated by AQS than RQS.

*Lactobacillus plantarum* inoculation altered the abundance of major bacteria involved in LA fermentation of alfalfa silage. These bacteria belong to the genera *Lactobacillus*, *Pediococcus*, *Weissella*, and *Leuconostoc* (Pang et al., 2011; Ni et al., 2018). *Lactobacillus* had become the predominant genus of the bacterial community in the inoculated group at 10day. While 10-day silage in the CK group exhibited a complex bacterial community composition. *Pediococcus* was the predominant genus in silage in the CK group at 60days. It is generally considered that LA-producing cocci were less competitive than *Lactobacillus* under low pH condition in silage (pH < 4.2). Yang et al. (2019) reported an increase on *Pediococcus* at early stage of alfalfa ensiling, and *Pediococcus* was later outcompeted by *Lactobacillus* under low pH condition. The pH condition in the 60d_CK silage (5.31) was still high, limiting the cocci metabolic inhibition and explaining the dominance of *Pediococcus* at 60days in the CK group. *Lactobacillus* predominated the bacterial community in the inoculated silage. This might be due to *L. plantarum* inoculant outcompeting other microorganisms. *Lactobacillus plantarum* inoculation accelerated LA fermentation, and thus restricted the proteolysis activity and the growth of undesirable microorganisms. This was also confirmed by the inhibition on NH_3_-N formation in the inoculated silage. Absolute abundance of *Lactobacillus* was reduced in the inoculated group at 60days compared with 10-day silage. This is consistent with reduction in LAB count with prolonged ensiling time reported by Li et al. (2018), Ren et al. (2019), and Wang et al. (2020) in alfalfa, oat, and sugarcane top silage, respectively. One reason for this reduction might be the decreased metabolic activity of *Lactobacillus* due to lack of fermentation substrates (Wang et al., 2020).

Although AQS and RQS both identified *Lactobacillus* and *Weissella* as high-dimensional biomarkers in microbiota among each treatment, AQS and RQS illustrated significant difference of these two genera on bacterial abundance in different treatments. This was caused by the incomprehensive interpretation of RQS due to the absence of absolute bacterial abundance determination. Relative abundance of these two genera increased at 60days compared with 10-day silage. But this increase was caused by the reduction in the absolute abundance of other bacterial groups.

### Correlation Analyses of the Bacterial Community With Fermentation Properties

Correlation analyses illustrated a negative correlation of bacterial diversity and fermentation quality. This is based on general recognition that LAB predominate the bacterial community and produce organic acids and mainly LA leading to pH decline during ensiling. Diversity of bacterial community will be reduced, as growth of other microorganisms is inhibited along with dominance of LAB. The low pH level in the inoculated silage inhibited the activities of plant proteolytic enzymes, which contributed to NH_3_-N inhibition. The WSC had negative correlations with richness of bacterial community. This is predictable, because WSC is a main nutrient source of microorganisms in silage and is thus consumed with the growth of microbes.

Relationships between bacterial genera and silage properties were also illustrated. Spearman’s correlation analyses indicated positive effects of *Lactobacillus* on fermentation quality of alfalfa silage. This is based on general recognition that *Lactobacillus* is a main producer of LA and plays an important role on pH reduction during ensiling (Cai et al., 1998). LA accumulation in silage leads to pH decline and inhibits growth of proteolytic microbes. LA-producers, including *Pediococcus*, *Enterococcus*, *Weissella*, and *Lactococcus*, exhibited negative correlations with fermentation quality. These genera were mainly observed in the CK group. These results were in accordance with former studies reported by Ogunade et al. (2018), Yang et al. (2019), and Dong et al. (2020). Hetero-fermentative LAB like *Weissella* are less efficient in decreasing pH than homofermentative *Lactobacillus*. *Hafnia* is a genus belonging to Enterobacteriaceae. It consumed nitrogen sources in silage and transformed them into alkaline products like biogenic amines and other NH_4_^+^ compounds, which increased pH level in silage. The relatively high abundance of *Weissella* and *Hafnia* might partly explain the higher pH level in the 10d_CK silage comparing with the 10d_LP silage. *Arthrobacter* also correlated positively with pH and WSC (*p* < 0.05). *Arthrobacter* are commonly found in aerial surface of plants with highly proteolytic activity (Gobbetti and Rizzello, 2014). This aerobe was observed at 10days but it was outcompeted by other microorganisms at 60day in the CK group.

The top tenth abundant genera illustrated by AQS and RQS varied. Because proportion of a certain genus in total abundance of all samples evaluated by AQS was based on its absolute abundance, while RQS evaluated the proportion based on its relative abundance in each sample. RQS dismissed variation in absolute abundance across samples. For instance, proportion of *Carnobacterium* was the ninth highest abundant genus observed in the total bacterial community evaluated by AQS, while its relative abundance was only ranked 12th through RQS analyses. Carnobacteria are ubiquitous heterofermentative LA producing bacteria isolated from cold and temperate environments. The genus was found to have antibacterial properties, and carnobacterial bacteriocins were applied toward inhibition of *Listeria* (Leisner et al., 2007). *Carnobacterium* was mainly observed in the non-treated silage at 10days (1.54×10^6^ copies/ng DNA), while its absolute abundance was reduced after 60-day ensiling (3.62×10^5^ copies/ng DNA). As observed for *Pediococcus*, *Enterococcus*, *Weissella*, and *Lactococcus*, AQS illustrated a negative correlation of *Carnobacterium* with silage fermentation quality.

Application of AQS bring deeper and more accurate information on bacterial community composition across samples, and aid in understanding the inner correlations between bacterial community and fermentation properties. To our knowledge, this is the first report of applying AQS in alfalfa ensiling research. Yeasts and molds are also important members on silage and their development strongly impact silage quality. With artificially designed synthetic chimeric DNA spikes representing 18S rRNA fragments with variable regions lacking identity to nucleotide sequences deposited in public databases, AQS may also be applied to fungi community analyses.

## Conclusion

*Lactobacillus plantarum* inoculation accelerated LA fermentation, inhibited NH_3_-N accumulation in alfalfa silage, and contributed to the rapid dominance of *Lactobacillus* in bacterial community. AQS provided more accurate information on bacterial community than RQS as it is based on absolute abundance, and effectively illustrated a more comprehensive dynamics of bacterial communities during ensiling. Application of AQS, thus, would aid in exploring dynamics of silage fermentation process.

## Data Availability Statement

The datasets presented in this study can be found in online repositories. The names of the repository/repositories and accession number(s) can be found in the article/supplementary material.

## Author Contributions

FY: conceptualization, formal analysis, writing – original draft, and visualization. SZ: methodology, formal analysis, validation, and investigation. YuW: resources and methodology. XF: resources. YaW: writing – review and editing, supervision, project administration, and funding acquisition. CF: writing – review and editing, supervision, and project administration.

### Conflict of Interest

The authors declare that the research was conducted in the absence of any commercial or financial relationships that could be construed as a potential conflict of interest.
